# Identification of multiple isomeric core chitobiose–modified high-mannose and paucimannose *N*-glycans in the planarian *Schmidtea mediterranea*

**DOI:** 10.1074/jbc.RA117.000782

**Published:** 2018-02-23

**Authors:** Sabarinath Peruvemba Subramanian, Ponnusamy Babu, Dasaradhi Palakodeti, Ramaswamy Subramanian

**Affiliations:** From the ‡ Institute for Stem Cell Biology and Regenerative Medicine (inStem), GKVK Post Office, Bellary Road, Bangalore 560065, Karnataka, India and; § Glycomics and Glycoproteomics Facility, Centre for Cellular and Molecular Platforms (C-CAMP), GKVK Post Office, Bellary Road, Bangalore 560065, Karnataka, India

**Keywords:** N-linked glycosylation, invertebrate, mass spectrometry (MS), galactosyltransferase, antisense RNA, Galβ1–4Fuc, GALT-1, O-methylation, planaria, S. mediterranea

## Abstract

Cell surface–associated glycans mediate many cellular processes, including adhesion, migration, signaling, and extracellular matrix organization. The galactosylation of core fucose (GalFuc epitope) in paucimannose and complex-type *N*-glycans is characteristic of protostome organisms, including flatworms (planarians). Although uninvestigated, the structures of these glycans may play a role in planarian regeneration. Whole-organism MALDI-MS analysis of *N*-linked oligosaccharides from the planarian *Schmidtea mediterranea* revealed the presence of multiple isomeric high-mannose and paucimannose structures with unusual mono-, di-, and polygalactosylated (*n* = 3–5) core fucose structures; the latter structures have not been reported in other systems. Di- and trigalactosylated core fucoses were the most dominant glycomers. *N*-Glycans showed extensive, yet selective, methylation patterns, ranging from non-methylated to polymethylated glycoforms. Although the majority of glycoforms were polymethylated, a small fraction also consisted of non-methylated glycans. Remarkably, monogalactosylated core fucose remained unmethylated, whereas its polygalactosylated forms were methylated, indicating structurally selective methylation. Using database searches, we identified two potential homologs of the Galβ1–4Fuc–synthesizing enzyme from nematodes (GALT-1) that were expressed in the prepharyngeal, pharyngeal, and mesenchymal regions in *S. mediterranea.* The presence of two GALT-1 homologs suggests different requirements for mono- and polygalactosylation of core fucose for the formation of multiple isomers. Furthermore, we observed variations in core fucose glycosylation patterns in different planarian strains, suggesting evolutionary adaptation in fucose glycosylation. The various core chitobiose modifications and methylations create >60 different glycoforms in *S. mediterranea.* These results contribute greatly to our understanding of *N*-glycan biosynthesis and suggest the presence of a GlcNAc-independent biosynthetic pathway in *S. mediterranea.*

## Introduction

Carbohydrates (glycans) on the cell surface are essential for cellular interactions. They mediate diverse cellular processes, such as adhesion, migration, signaling, extracellular matrix organization, development, host–pathogen interactions, and immunity (1). Glycans are heterogeneous molecules; they vary widely in their composition, structure, distribution, linkage, stereochemical organization, and covalent modifications. Several studies have demonstrated that glycans are expressed in a spatiotemporal (2, 3) and species-specific manner (4) determined by levels of glycosyltransferases/glycosylhydrolases and availability of nucleotide-sugar donors and acceptors. This selectivity in expression pattern is a key determinant of the roles they play in cellular interactions. Alterations in cell-surface glycans can modify cellular function; some aberrant alterations can lead to embryonic lethality and developmental abnormalities (5). Therefore, it is accepted that glycoproteins and glycolipids play a major role in cell fate determination, including differentiation and development. However, our understanding of the mechanisms by which glycans act is limited. To address this, several studies have used *in vitro* cell-based systems and vertebrate and invertebrate models to understand the role of glycans in development and cellular transformation (6–8). Although genetically amenable, commonly used model systems, such as mice, *Caenorhabditis elegans*, and *Drosophila melanogaster* have a rigid or complex developmental process. Because glycans themselves are highly complex, using these systems to study the role of glycans in various processes becomes very difficult. This necessitates the use of simpler and more plastic model systems like planarians.

Planarians are of particular interest in stem cell biology and regenerative medicine because of their remarkable cellular plasticity and immense regenerative potential. A small body fragment can regenerate all body parts, regrow, and resize into a fully functional organism. In contrast to other invertebrate models, planarians can constantly replenish terminally differentiated organs and precisely restore their body plan. This property is attributed to the presence of pluripotent stem cells named “neoblasts” (9). Although planarians lack a body cavity as well as skeletal, respiratory, and circulatory systems, they do have digestive (10), nervous (11), muscular (12), reproductive (13), and excretory systems (14). Planarians exist as both sexual and asexual strains. Sexual strains are hermaphrodites; they cross-fertilize and lay eggs that undergo complex, anarchic embryogenesis (15), whereas asexual strains propagate via transverse fission. In the last two decades, genome and transcriptome analyses combined with the use of molecular tools, such as RNA interference (16) and *in situ* hybridization (17), have led to the identification of several genes essential for homeostasis, regeneration, and patterning in planarians. However, little is known about the role of post-translational events, specifically glycosylation, in mediating these biological processes. Therefore, the structural characterization of glycans is a logical first step in understanding the role of glycans in regeneration and tissue homeostasis in planarians. Previous studies by Natsuka *et al.* (18) and Paschinger *et al.* (19) have characterized the *N*-glycome of the planarian *Dugesia japonica*. In this study, we characterize the *N*-glycome of the diploid, sexual strain of *Schmidtea mediterranea*, which is currently one of the most widely used planarian species. The *N*-glycans from *S. mediterranea* showed unusual structural complexity in their multiplicity, core chitobiose modifications, and methylation patterns. Furthermore, our work also demonstrates that the glycomes of different species of planarians are unique. These findings reiterate that invertebrates do not have simplistic glycomes and that these glycomes are derived from a complex biosynthetic process.

## Results

### Total N-glycome profile of S. mediterranea

**Figure 1.**
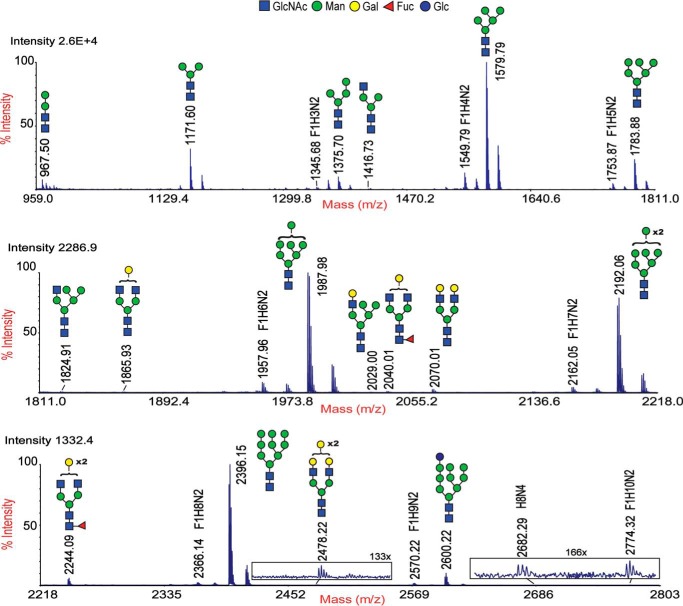
**MALDI-TOF MS spectra of permethylated *N*-glycans from *S. mediterranea*.** Shown are MS spectra of the 50% acetonitrile fraction of permethylated *N*-glycans obtained from the C_18_ Classic cartridge. Molecular ions indicated are ^12^C monoisotopic peaks of singly charged monosodiated [M + Na]^+^ *N*-glycans released with PNGase A treatment. The structures were annotated based on the putative composition, MS/MS fragmentation pattern, and biosynthetic knowledge and represented according to the symbols used in the consortium for functional glycomics. Glycans at peaks of *m*/*z* 1345, 1549, 1753, 1957, 2162, 2366, and 2570 with multiple isomers are represented by their putative compositions: fucose (*F*), hexose (*H*), and HexNAc_2_ (*N*). Data presented here are representative of the MS spectra of three biological trials. Mass accuracy, ±0.05 Da. Monosaccharide moieties represented outside the *brackets* have not been unequivocally defined.

**Figure 2.**
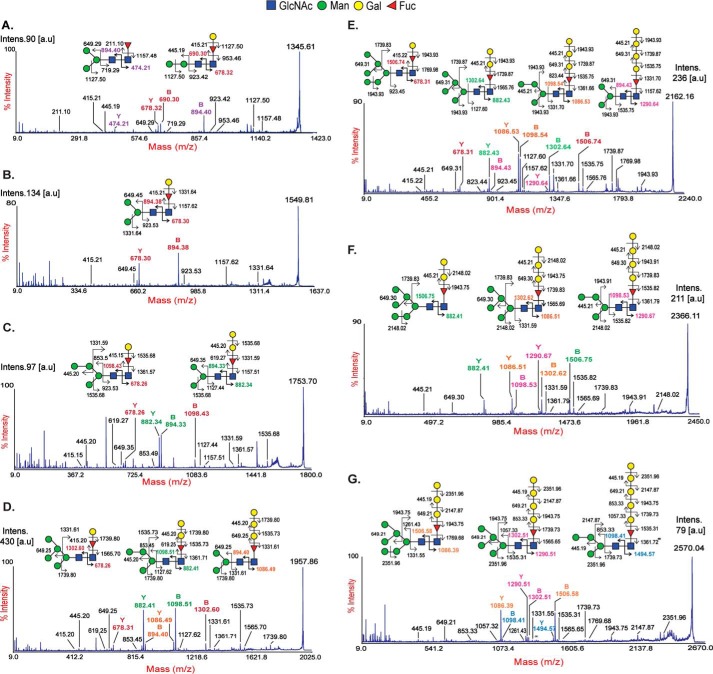
**MALDI-TOF/TOF and MS/MS spectra of the Fuc_1_Hex*_n_*HexNAc_2_ series.** Major peaks corresponding to the F_1_H*_n_*N_2_ series were subjected to MALDI-TOF/TOF analysis. Shown are MS/MS spectra of singly charged monosodiated [M + Na]^+^ permethylated *N*-glycans of *m*/*z* 1345 (*A*), 1549 (*B*), 1753 (*C*), 1957 (*D*), 2162 (*E*), 2366 (*F*), and 2570 (*G*) obtained in the 50% acetonitrile fraction. The B- and Y-fragment ions obtained are represented. Major B/Y-ion pairs corresponding to each isomer are color-matched. Major B-ions of *m*/*z* 690, 894, 1098, 1302, and 1506 correspond to Man_2–6_GlcNAc structures at the non-reducing termini. The Y-ion of *m*/*z* 474 represents fucose linked to proximal GlcNAc. The Y-ions of *m*/*z* 678, 882, 1086, 1290, and 1494 correspond to mono- or polygalactosylated fucose linked to reducing terminal core GlcNAc. The data presented here are representative of the MS/MS spectra of three biological trials. *Intens*., intensity; *a.u.*, arbitrary units.

Ultrasensitive MALDI-TOF (MS) and TOF/TOF (MS/MS) techniques were used to characterize PNGase A^3^–released *N*-glycans from whole planarian. The *N*-glycome of *S. mediterranea* was primarily composed of paucimannose (*m*/*z* 967.5, 1171.6, and 1375.7) and high-mannose structures (*m*/*z* 1579.8, 1783.9, 1988.0, 2192.0, 2396.1, and 2600.2) as well as structures having compositions of Fuc_1_Hex_2–9_HexNAc_2_ (F_1_H*_n_*N_2_) (*m*/*z* 1345.7, 1549.8, 1753.9, 1957.9, 2162.0, 2366.1, 2570.2, and 2774.3). Additionally, low amounts of complex-type (*m*/*z* 1416.7, 1865.9, 2040.0, 2070.0, 2244.1, 2478.2, and 2682.3) and hybrid-type (*m*/*z* 1824.9 and 2029.0) glycans were also observed (Fig. 1). The MS/MS analyses of individual masses in the F_1_H*_n_*N_2_ series revealed that each mass was composed of multiple isomers (glycomers), evident from the presence of two or more major B- and Y-ions formed from the breakdown of the facile glycosidic bond between the core GlcNAcs of a specific oligosaccharide motif (Fig. 2, *A–G*). Major B-type ions (*m*/*z* 690, 894, 1098, 1302, and 1506) formed the non-reducing termini with compositions corresponding to Hex_2–6_GlcNAc_1_, whereas major Y-type ions (*m*/*z* 474, 678, 882, 1086, 1290, and 1494) formed the reducing termini with compositions corresponding to Fuc_1_Hex_0–5_GlcNAc_1_. There was a tendency for the number of glycomers within a given mass to increase as the molecular mass increased. However, this tendency was observed only until *m*/*z* 2162.0 for which a maximum of four glycomers was observed. Subsequently, for *m*/*z* 2366.1 and 2570.2, only three glycomers were observed. The abundance of individual glycomers within a mass was variable; di- and trihexosylated core fucose was the most predominant isomer followed by mono-, tetra-, and pentahexosylated core fucose (Fig. 2, *A–G*, and Table 2). Complex-type *N*-glycans with core fucose of *m*/*z* 2040.0 and 2244.1 also showed the presence of core chitobiose modifications (CCMs) with mono- and dihexosylated core fucose. MS/MS spectra of complex and hybrid structures are presented in Fig. S1.

### Unusual core chitobiose–modified structures are glycomers of mono- and polygalactosylated core fucose

**Figure 3.**
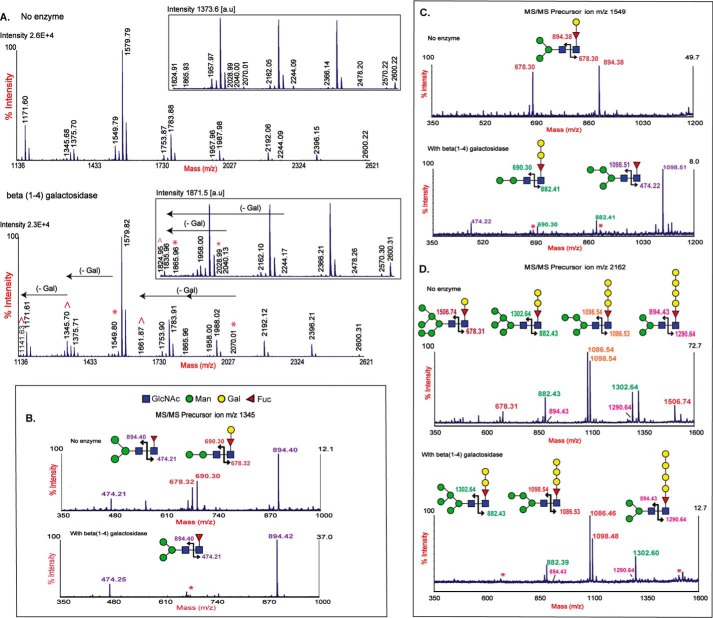
**MALDI-TOF MS and MS/MS spectra of β(1–4)-galactosidase-treated *N*-glycans.** PNGase A–released *N*-glycans treated with and without β(1–4)-galactosidase (from *A. oryzae*) were permethylated and analyzed using MALDI-TOF MS and MALDI-TOF/TOF MS/MS. *A*, MS spectra of singly charged monosodiated [M + Na]^+^ permethylated *N*-glycans in galactosidase-treated and -untreated *N*-glycans. Glycans susceptible to *Aspergillus* β(1–4)-galactosidase treatment lost a galactose residue (204 Da) and showed a concomitant reduction in peak intensities at *m*/*z* 1549, 2040, 2070, 2244, and 2029. Loss of the galactose residue resulted in increases in peak intensities at *m*/*z* 1141, 1345, 1161, 1824, and 1836 (> indicates increased peak intensity, * indicates reduced peak intensity, and ← indicates change between original *m*/*z* and products formed after enzyme treatment). Other CCM structures of *m*/*z* 1957, 2162, 2366, and 2570 were resistant to β(1–4)-galactosidase. *B–D*, MS/MS spectra of *m*/*z* 1549, 1753, and 2162. Following β(1–4)-galactosidase treatment, MS/MS data show loss of Y-ion *m*/*z* 678 and its corresponding B-ion (*). Loss of a hexose from *m*/*z* 678 indicates the presence of a galactose 4-linked to core fucose. Other Y-ions of *m*/*z* 882, 1086, 1290, and 1496 remained resistant to β(1–4)-galactosidase. *a.u.*, arbitrary units.

Based on the characteristic B/Y-ions, it was inferred that the glycomers were composed of paucimannose and high-mannose (Man-2 to Man-6) structures with unusual CCM. To ascertain the monosaccharide composition and linkage of the CCM structures, *N*-glycans were subjected to chemical/enzymatic hydrolysis and methylation analysis. No intensity changes were observed in peaks of the F_1_H*_n_*N_2_ series after hydrofluoric acid (specific to α(1–3)- and α(1–2)-fucose) and α-fucosidase (from bovine kidney) treatment as compared with those from untreated sample (Fig. S2, A–C). This result indicates that fucose moieties are α(1–6)-linked to core GlcNAc (reducing termini) and are capped by hexoses. As galactosylated fucose moieties are reported to be constituents of CCMs in *N*-glycans of protostomes (20), we suspected that the hexoses attached to core fucose were galactose. To confirm this, these structures were subjected to β(1–4)-galactosidase (from *Aspergillus oryzae*) treatment. Although a significant reduction in the intensity of *m*/*z* 1549.8 with a concomitant increase in the intensities of *m*/*z* 1345.6 and 1141.6 was seen, no significant changes were observed in the intensities of peaks of *m*/*z* 1753.8, 1957.9, 2162.0, 2366.1, and 2570.2 (Fig. 3*A*). Interestingly, MS/MS analysis following β(1–4)-galactosidase treatment revealed that only Y-ion *m*/*z* 678 was susceptible to β(1–4)-galactosidase, whereas Y-ions *m*/*z* 882, 1086, 1290, and 1494 were resistant (Fig. 3, *B–D*). The loss of galactose from Y-ion *m*/*z* 678 resulted in Y-ion 474 and a concomitant shift to the preceding mass. This result confirmed that Y-ion *m*/*z* 678 was composed of Galβ1–4Fuc motif (GalFuc) and formed the core of Man-2 to Man-6 structures. As other major Y-ions were resistant to β(1–4)-galactosidase treatment, we tested for alternate linkages of galactose. Treatment with β(1–3/6)-galactosidase (from *Xanthomonas manihotis*; highly reactive to 3-linked galactose and moderately reactive to 6-linked galactose) and α-galactosidase (from coffee beans) did not show any change in the F_1_H*_n_*N_2_ series (Fig. S2, D and E). This eliminated the presence of 3- and 6-linked as well as α-linked galactose. In contrast, monosaccharide and linkage analyses using gas chromatography-mass spectrometry (GC-MS) showed the presence of terminal galactose, 4-linked galactose, 4-linked fucose, and 4,6-linked *N*-acetylglucosamine residues (Table 1 and Fig. S3). The 1:8 ratio of terminal galactose to 4-linked galactose confirmed that the unusual CCM structures were composed of extensions of 4-linked galactose (polygalactosylated core fucose). However, the anomer specificity of the polygalactosylated core fucose could not be established as both α- and β-galactosidase were ineffective in hydrolyzing these core-modified structures. Thus, the structures of the multiple isomers with CCM seen in the F_1_H*_n_*N_2_ series were established to be Man-2 to Man-6 containing Galβ1–4Fucα1–6GlcNAc and poly-Galα/β1–4Fucα1–6GlcNAc at the core reducing termini.

**Table 1:** **Monosaccharide composition and linkage analysis of N-linked oligosaccharides in S. mediterranea** PNGase A–released glycans were permethylated, hydrolyzed, reduced, and acetylated. PMAAs thus formed were subjected to GC-MS analysis.

Elution time*^a^*	Signature ions*^a^*	Linkage assignment
*min*		
09.51	101, 118, 143, 203	4-Linked fucose
09.92	102, 118, 129, 145, 161, 205	Terminal mannose
10.15	102, 118, 129, 145, 161, 205	Terminal galactose
10.96	129, 130, 161, 190	2-Linked mannose
11.18	113, 118, 131, 173, 233	4-Linked galactose
11.29	118, 129, 161, 234, 277	3-Linked mannose
11.42	102, 118,129, 162, 189	6-Linked mannose
12.55	118, 129, 189, 234	3,6-Linked mannose
14.25	117, 159, 233	4-Linked *N*-acetylglucosamine
15.48	117, 159, 261	4,6-Linked *N*-acetylglucosamine

*^a^* GC chromatogram and MS spectra of PMAAs (Fig. S3). Terminal fucose was undetected.

### N-glycans in S. mediterranea are selectively methylated

**Figure 4.**
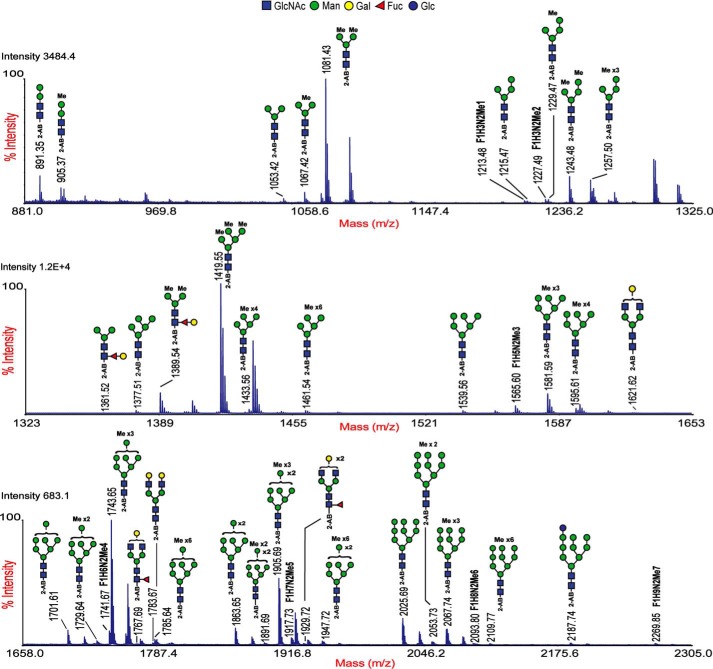
**MALDI-TOF MS spectra of 2-AB-labeled *N*-glycans from *S. mediterranea*.** Shown are MS spectra of 2-AB–labeled *N*-glycans obtained in the 50% acetonitrile fraction using a Hypercarb cartridge. The molecular ions represented here are ^12^C monoisotopic peaks of singly charged monosodiated [M + Na]^+^ 2-AB–labeled *N*-glycans released with PNGase A treatment. The structures of the glycans were annotated based on the MS/MS data and knowledge of biosynthesis and depicted according to the symbols used in the consortium for functional glycomics. The masses of the annotated peaks show consecutive increases of 14 Da, indicating extensive methylation. Glycans with a putative composition belonging to the Fuc_1_(*F*)Hex*_n_*(*H*)HexNAc_2_(*N*) series are represented in characters, and *Me* indicates methylation. The data presented here are representative MS spectra of three biological trials. Mass accuracy, ±0.05 Da. Monosaccharide moieties and methyl groups (*Me*) represented outside the *brackets* are not unequivocally defined.

**Figure 5.**
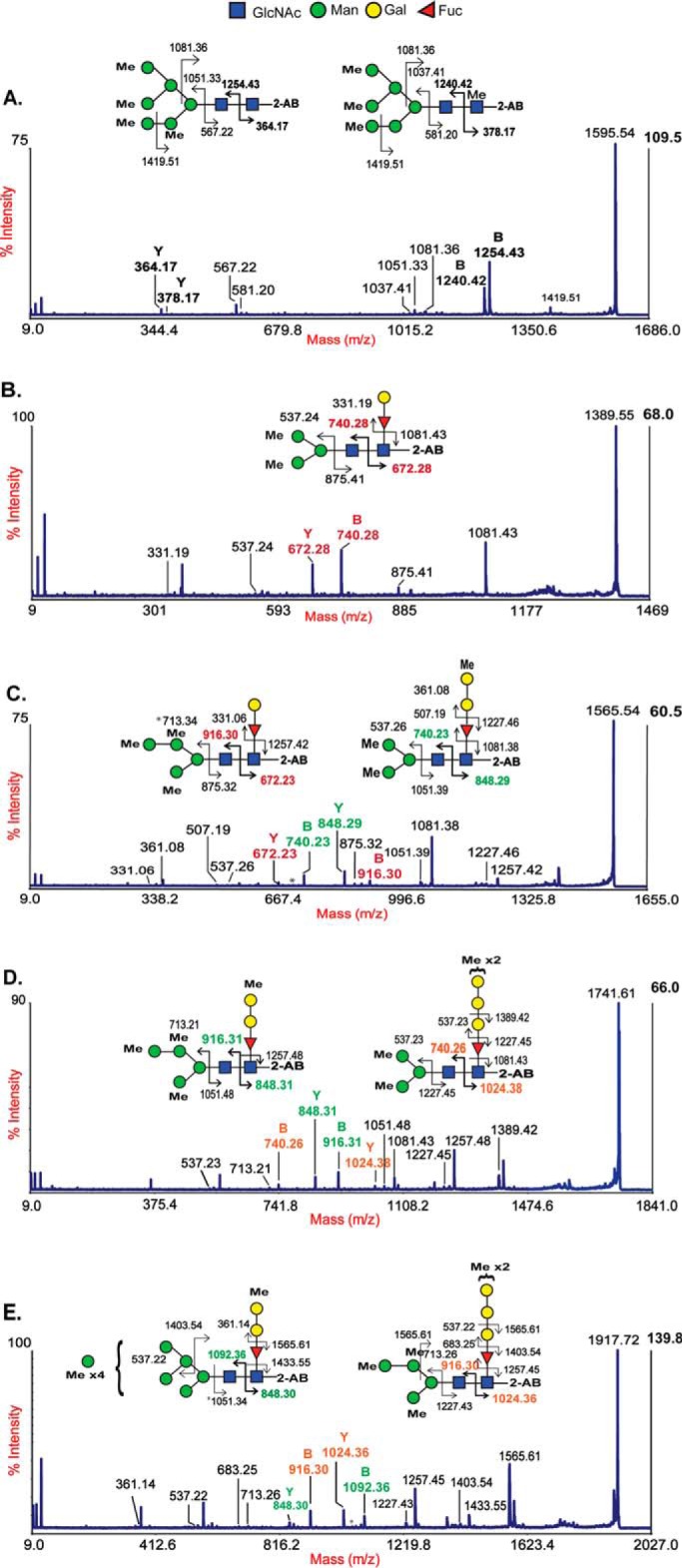
**MALDI-TOF MS/MS spectra of 2-AB-labeled glycans.** The data represent MS/MS spectra of singly charged monosodiated [M + Na]^+^ 2-AB–labeled *N*-glycans released with PNGase A treatment. *A*, MS/MS of *m*/*z* 1565. The B-ions of *m*/*z* 1240 and 1254 represent the presence of two isomers with differences in methylation along the non-reducing mannose termini. The Y-ions *m*/*z* 364 and 378 differing by 14 Da indicate the methylated and non-methylated core. *B–E*, MS/MS spectra of CCM structures of *m*/*z* 1389, 1565, 1741, and 1917. The Y-ion of *m*/*z* 672, corresponding to GalFuc in the parent mass 1389, shows no methylation, whereas the Y-ions of *m*/*z* 848 and 1024 represent the sequential addition of methyl groups (+14 Da) in the extended GalFuc structures. The positions of methyl groups (*Me*) on the mannose and galactose moieties are not unequivocally defined and are represented outside the *brackets*.

Despite the presence of terminal and 4-linked galactose in CCM, its resistance to galactosidase was puzzling. A possible explanation for this phenomenon could be methylation as methylated glycans have been shown to be resistant to enzymatic digestion (18). To verify this, *N*-glycans were subjected to 2-aminobenzamide (2-AB) labeling and mass spectrometry. MALDI-TOF MS analysis revealed the presence of both non-methylated and methylated *N*-glycans with a greater abundance of methylated glycans (Fig. 4). Paucimannose and high-mannose glycans displayed diverse methylation patterns, spanning from monomethylated to polymethylated forms (having up to six methyl groups) among which trimethylated species (*m*/*z* 1419.5, 1581.6, 1743.6, 1905.7, and 2067.7) were the most predominant. MS/MS analysis of methylated high mannose–type structures revealed that methylation occurred at both the mannose termini as well as at the core GlcNAc residues (Fig. 5*A* and Fig. S4), giving rise to isomers of methylated glycans. Furthermore, α-mannosidase (from jack bean) treatment resulted in the hydrolysis of non-methylated glycans, whereas methylated glycans were resistant to this treatment. In addition to confirming the presence of methylation, these results also indicated that methylation occurred at the terminal mannose residues, explaining the resistance to α-mannosidases (Fig. S5). MS/MS analysis of CCM structures revealed that only the Y-ion (*m*/*z* 672) corresponding to Galβ1–4Fuc was devoid of methylation. Other Y-ions (*m*/*z* 848, 1024, and 1200) corresponding to polygalactosylated core fucose structures showed successive increases of 14 Da for every additional galactose (Fig. 5, *B–E*). Because the extended CCM structures showed resistance to both α-mannosidase and β(1–4)-galactosidase digestion, we deduced that the mannose termini and the extended galactose termini in F_1_H*_n_*N_2_ are methylated. These results confirm the presence of methylation in CCM structures and explain the resistance of the polygalactosylated core fucose structures to β(1–4)-galactosidase. Other glycan modifications, such as phosphorylation of mannose and phosphorylcholine on GlcNAc moieties, were not observed.

### Identification and distribution of galt-1 in S. mediterranea

**Figure 6.**
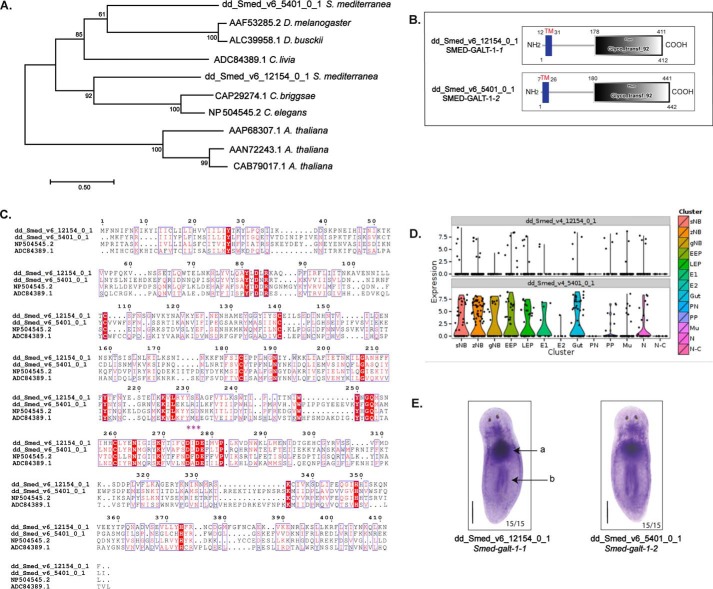
**Identification and expression of GALT-1 in *S. mediterranea*.** *A*, maximum likelihood phylogenetic tree of identified SMED-GALT-1 proteins. The phylogenetic tree was generated from select sequences belonging to the GT-92 family. *B*, the domain architecture of SMED-GALT-1 as visualized using SMART. The domain structure indicates the presence of a transmembrane domain (*TM*) at the N terminus and a GT-92 (*Glyco_transf_92*) domain at the C terminus. *C*, multiple sequence alignment of SMED-GALT-1 hits (dd_Smed_v6_12154_0_1 and dd_Smed_v6_5401_0_1) and *C. elegans* (NP_504545.2) and *C. livia* (ADC84389.1) protein sequences. Sequences were aligned using MUSCLE and presented using ESPript 3.0 (57). The SMED-GALT-1 sequences show 26% identity with the *C. elegans* and *C. livia* homologs. The * represents the D*X*D domain, which is characteristic of Mn^2+^-dependent glycosyltransferases. *D*, the distribution of putative *Smed-galt-1* homologs in different cell types using the SCS database (*sNB*, σ neoblast; *zNB*, ζ neoblast; *gNB*, γ neoblast; *EEP*, early epidermal progenitor; *LEP*, late epidermal progenitor; *E1*, epidermis 1; *E2*, epidermis 2; *Gut*, intestine; *PN*, protonephridia; *PP*, parapharyngeal; *Mu*, muscles; *N*, neural; *N-C*, ciliated neurons). *E*, whole-mount *in situ* hybridization representing the spatial distribution of *galt-1* in *S. mediterranea. Smed-galt-1* is expressed in the prepharyngeal region (*a*), pharynx (*b*), and mesenchyme (*c*), the loose undefined tissue within the organism. *Scale bar*, 200 μm.

As galactosylation of core fucose was the predominant modification observed in the *N*-glycans from *S. mediterranea*, we hypothesized the presence of homologs of *C. elegans* GALT-1 (known to catalyze galactosylation of core α(1–6)-fucose (Galβ1–4Fuc)) (21) in the organism. We used the GALT-1 sequence from *C. elegans* (NP_504545.2) and its avian (*Columba livia*) homolog (FJ971845.1/ADC84389.1; shown to catalyze galactosylation of terminal β(1–4)-galactose in the antennae of complex-type *N*-glycans) (22) as query sequences to identify putative GALT-1 sequences in *S. mediterranea.* Sequence similarity searches using BLASTp in the PlanMine database (23) revealed the presence of two putative GALT-1 sequences, a short dd_Smed_v6_12154_0_1 (SMED-GALT-1-1) sequence and a long dd_Smed_v6_5401_0_1 (SMED-GALT-1-2) sequence. Phylogenetic analyses showed SMED-GALT-1-1 to be a homolog of the nematode GALT-1 and SMED-GALT-1-2 to be a homolog of the arthropod GALT-1 (Fig. 6*A*). A complete phylogenetic tree with all GALT-1 entries listed in the CAZy database along with SMED-GALT-1 is provided in Fig. S6. The two SMED-GALT-1 homologs showed 22–26% identity with the GALT-1 sequences from *C. elegans* and *C. livia* and exhibited a characteristic type II transmembrane domain, a GT-92 domain, and a D*X*D motif, signifying potential catalytic activity (Fig. 6, *B* and *C*). To determine the spatial distribution of *galt-1* homologs, two methods were used: (*a*) bioinformatics analysis using a single cell sequencing (SCS) database (24) and (*b*) whole-mount *in situ* hybridization. The SCS database search revealed that the expression of dd_Smed_v6_12154_0_1 (*Smed-galt-1-1*) was sparse, whereas the expression of dd_Smed_v6_5401_0_1 (*Smed-galt-1-2*) was predominant in the neoblast, epidermal cells, gut, and neurons (Fig. 6*D*) of *S. mediterranea.* Contrary to this, whole-mount *in situ* hybridization showed that both homologs of *Smed-galt-1* were expressed in the prepharyngeal region, pharynx, and mesenchyme (Fig. 6*E*) of *S. mediterranea.* These results corroborate the presence of two putative GALT-1 enzymes that may be involved in the mono- and polygalactosylation of core fucose structures. To ascertain the role of these putative candidates in tissue homeostasis, the target genes were knocked down using RNA interference (RNAi) technology with double-stranded RNA (dsRNA). Knockdown of *Smed-galt-1* resulted in a 60–70% reduction in RNA expression as compared with the controls (Fig. S7A). Knockdown using RNAi resulted in the occurrence of an unusual stick and stretch phenotype (Fig. S7B), suggesting an alteration in tissue homeostasis.

## Discussion

The occurrence of galactosylated fucose structures at the core of *N*-glycans is characteristic of protostomes and has been observed previously in various organisms, such as octopus (25), squid (26), snail (27, 28), nematode (29), and planarian (19). This modification, which includes both mono- and digalactosylated core fucose, has only been observed in paucimannose and complex- monoantennary-type structures. In this study, we show, for the first time, that CCM structures are not restricted to paucimannose and complex-type *N*-glycans. Rather, they span across Man-2 to Man-6 structures (possibly even Man-7 in *m*/*z* 2774.3) in an extended fashion ranging from mono- to polygalactosylated core fucose (poly-Gal_1–5_Fuc) (Fig. 2 and Table 2). However, it is possible that B-ions *m*/*z* 1098 and 1302 corresponding to Hex_4_GlcNAc_1_ and Hex_5_GlcNAc_1_ may be paucimannosidic structures with terminal galactose residues. These glycans have been observed in wildtype, pseudo-wildtype (*pmk-1* knockdown), and fucosyltransferase knockout *C. elegans* (30, 31). Unfortunately, the extensive methylation (causing resistance to enzymatic hydrolysis) noted here and insufficient material for NMR or GC-MS of individual glycan fractions observed here limits the validation of these above mentioned structures.

**Table 2:**
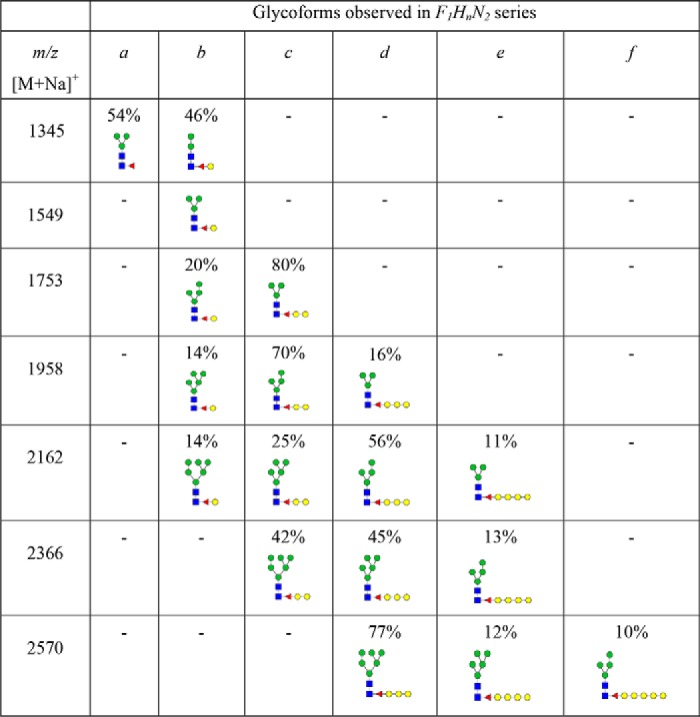
**Summary of glycomers observed in F_1_H_n_N_2_ series** Molecular ions represent singly charged monosodiated permethylated *N*-glycans in the F_1_H_n_N_2_ series observed in MALDI-TOF MS spectra (Fig. 1). Based on the extensions seen in the core chitobioses, different glycomers were grouped into forms ranging from a-form to f-form. Percentages represent relative distributions of each glycomer observed in the MALDI-TOF/TOF MS spectra (Fig. 2). The relative distribution of each glycomer was determined by calculating the intensities of major B- and Y-ion pairs corresponding to a particular glycoform and dividing it by the sum total of the intensities of all major Band Y-ions corresponding to each glycomer within the individual parent mass.

**Figure 7.**
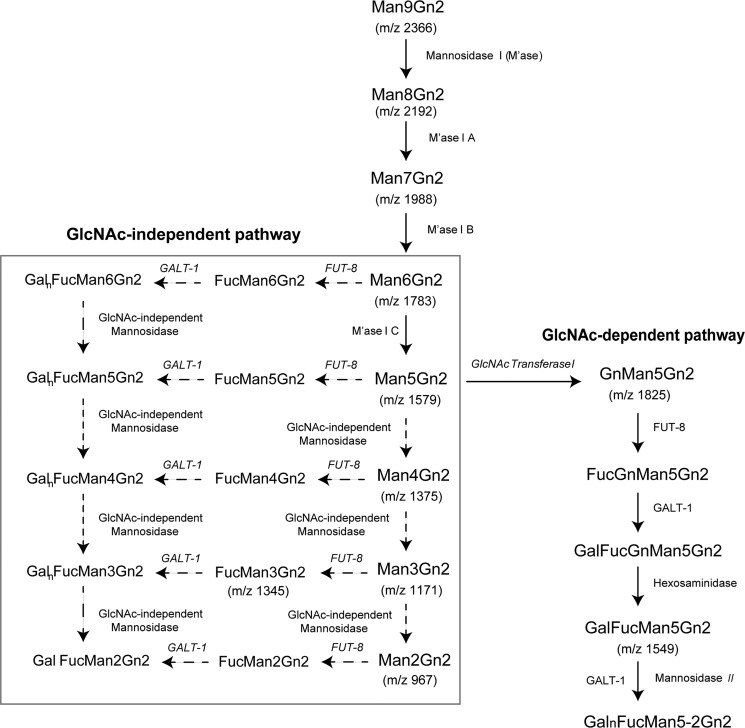
**Proposed biosynthetic scheme for the galactofucosylation of high-mannose and paucimannosidic structures.** MALDI-TOF and TOF/TOF MS analyses of both permethylated and 2-AB–labeled *N*-glycans reveal the presence of multiple isomeric structures containing mono- and polygalactosylated core fucose structures. The presence of high mannose with such modifications suggests the occurrence of a GlcNAc-independent fucosylation pathway. The proposed biosynthetic scheme for the production of the above mentioned structures would involve core fucosylation reactions independent of β(1–2)-GlcNAc at the α(1–3)-Man arm and subsequent galactosylation (via GALT-1-1 or GALT-1-2 (*) whose functions are currently unknown) and trimming by mannosidase (represented by the *dotted lines* and the *red box*). An alternate biosynthetic scheme would involve GlcNAc-dependent biosynthesis where Man-5 to Man-2 are formed through the sequential action of FUT-8, hexosaminidase, mannosidase II, and GALT-1. This biosynthetic scheme was constructed using the data on molecular ions obtained through MALDI-TOF MS spectra of PNGase A–released *N*-glycans. The molecular ions indicated are singly charged monosodiated permethylated *N*-glycans. *Gn*, *N*-acetylglucosamine.

Our analyses of these structural data have had an interesting consequence on our understanding of *N*-glycan biosynthesis. Based on the molecular ions obtained in MS spectra, we suspect a GlcNAc-independent biosynthetic pathway to be functional here. Although it has been demonstrated that the presence of β(1–2)-GlcNAc on the α(1–3)-Man arm is a prerequisite for α(1–6)-core fucosylation (FUT-8) and subsequent galactosylation (GALT-1), *i.e.* GalFuc formation (4, 32–34), the occurrence of GlcNAc-independent α(1–6)-core fucosylation has also been reported *in vitro* following knockout of GlcNAc-transferase-I (35–37). Notably, the presence of core-fucosylated high mannose has been reported in placental arylsulfatase A in humans (38). However, the mechanism of GlcNAc-independent core fucosylation still remains unclear. A recent report suggests that FUT-8 interacts with the appropriate protein or peptide regions (in erythropoietin and the V3 domain peptide) to facilitate core fucosylation of oligomannoses (39). The presence of Man-5 and Man-6 with mono- and polygalactosylated core fucose structures observed in this study suggests that these glycans may have been synthesized through a GlcNAc-independent core fucosylation pathway. These structures provide evidence for the natural occurrence of α(1–6)-core fucosylation and subsequent galactosylation of high mannose *in vivo* (Fig. 7). Alternatively, subsequent to core fucosylation, GlcNAc residues on the α(1–3)-Man arm can be truncated by hexosaminidase to form core chitobiose–modified Man-5. These structures can then undergo trimming by mannosidase to form Man-4, -3, and -2 with core modifications (via the GlcNAc-dependent pathway) (Fig. 7). The low levels of complex-type structures observed here support the presence of extensive hexosaminidase activity in this system. Therefore, it is likely that in planarians the biosynthesis of core chitobiose–modified *N*-glycans proceeds via both GlcNAc-dependent and -independent pathways.

So far, GALT-1 (belonging to the GT-92 family of enzymes from *C. elegans*) is the only enzyme known to add galactose to α(1–6)-core fucose. Two other enzymes from the GT-92 family have been shown to have different functions; one is the avian homolog of GALT-1 known to catalyze the addition of β(1–4)-galactose to complex biantennary structures (22), and the other is GALS1 (a β(1–4)-galactan synthase) from *Arabidopsis thaliana* that catalyzes Gal-Gal formation in pectin (40). Our results demonstrate the presence of two genes that belong to the GT-92 family in the planarian *S. mediterranea* (Fig. 6 and Fig. S6). Of the two, dd_Smed_v6_12154_0_1 (SMED-GALT-1-1), which is homologous to *C. elegans* GALT-1, is the most likely to have GALT-1–like activity. The other gene, dd_Smed_v6_5401_0_1 (SMED-GALT-1-2), is homologous to *Drosophila* GALT-1. However, because no “GalFuc” moieties have been reported in *Drosophila*, the GALT-1 function in *Drosophila* remains unknown. Given the diversity in enzyme function among the different members of the GT-92 family and in the galactosylation patterns in the core and termini in planarians, understanding the mechanisms of action of the SMED-GALT-1 orthologs in planarians can provide further insights into substrate specificity. The stick and stretch phenotype in *S. mediterranea*, observed following the knockdown of *Smed-galt-1*, has been reported earlier as occurring in knockdowns of the hepatocellular-associated carcinoma antigen (NBE 8.11C) (16). However, cellular alterations in this phenotype have not been characterized and warrant further investigation. Further characterization of this phenotype will also aid in identifying the role of GalFuc in planarian physiology.

This is the first study to perform a comprehensive analysis of methylated *N*-glycans in *S. mediterranea*, and our work brings to light a remarkable feature of the *N*-glycans in this system, their methylation patterns. In *D. japonica*, trimethylated structures were found to be the most predominant forms of *N*-glycans (18, 19). However, in *S. mediterranea*, significant levels of tetra-, penta-, and hexamethylated *N*-glycans with methylation at both mannose and GlcNAc termini have been observed. Furthermore, the selective sparing of monogalactosylated fucose from methylation (only polygalactosylated fucose structures were methylated at the core) in *S. mediterranea* was rather intriguing. The detection of methylated species also explained the resistance of *S. mediterranea N*-glycans to galactosidase digestion, although the position of methylation and the number of methyl groups on the monosaccharides could not be ascertained in this study. Besides this, little is known about the enzymes responsible for methylation in invertebrates as such enzymes have yet to be identified. Wohlschlager *et al.*(41) have demonstrated that knockout of *smt-1* (*S*-adenosylmethionine transporter) reduces glycan methylation in *C. elegans*, suggesting that methylation of glycans occurs in the Golgi body. The selective methylation seen here, however, suggests that methylating enzymes are specific. Additionally, planarians have been shown to resist bacterial strains that infect humans (42). This characteristic may be attributed to the extensive methylation of glycans seen in planarians as methylated glycans are known to prevent bacterial adhesion, thereby conferring resistance to bacterial infections.

This study also revealed considerable differences in the *N*-glycomes of different species and strains of planarians. The *N*-glycomes of *D. japonica* (18, 19), which is closely related to *S. mediterranea*, and an Indian strain of *Dugesia* (IN06) did not show the presence of higher masses with polygalactosylated core fucose structures and multiple isomers at the core (Fig. S8) as seen in *S. mediterranea.* These differences in the glycome profiles may be the result of differences in the glycogenomic potential of different planarian species. Determining the temporal and spatial distributions of these CCM structures and those of the methylated and non-methylated *N*-linked oligosaccharides using MALDI-based imaging and antibodies against GalFuc epitopes will contribute to a better understanding of their tissue-specific functions.

## Experimental procedures

### Materials

All chemicals used in this study were of analytical grade and purchased from Sigma-Aldrich unless otherwise mentioned.

### Planarian maintenance

Planarians were maintained in the dark at 20 °C in planarian medium (43) and fed with homogenized beef liver twice a week. Planarians were starved for 10 days prior to experiments.

### Glycan analysis: Preparation of glycopeptides and release of N-glycans

Glycopeptide extracts from whole-planarian tissues were prepared using surfactant-aided precipitation/on-pellet digestion (44). Planarians (100 mg wet weight) were homogenized with sonication in 4 ml of 50 mm phosphate-buffered saline, pH 7.4 (PBS), containing 1% SDS and protease inhibitor mixture (Roche Diagnostics) using a Q500 sonicator (Qsonica, LLC; at 20% amplitude with 20-s pulse and 10-s pause for 10 min), reduced (10 mm DTT for 30 min at 56 °C), and carboxymethylated (25 mm iodoacetic acid in the dark for 30 min at 37 °C). Carboxymethylated proteins were precipitated by adding 6 volumes of ice-cold acetone in a stepwise manner with vigorous mixing and incubation at −20 °C for 3 h. Precipitated proteins were collected by centrifugation at 20,000 × *g* for 30 min at 4 °C, washed (in 6:1 (v/v) acetone:ultrapure water), air-dried, and subjected to tryptic digestion (1:25 (w/w) enzyme:substrate) in 50 mm ammonium bicarbonate buffer, pH 8.5, for 16 h at 37 °C. Trypsinization was stopped by adding a few drops of acetic acid and heating at 100 °C for 3 min. Glycopeptides were purified on an Oasis® HLB cartridge (Waters) using a 5% acetic acid, propanol solvent system. *N*-Glycans from glycopeptides were released by incubating with 10 μl of PNGase A (Roche Applied Science) in ammonium acetate buffer, pH 5.0, for 24 h at 37 °C. Liberated glycans were recovered in the 5% acetic acid fraction using a Sep-Pak Classic C_18_ cartridge (Waters) after which they were lyophilized and subjected to permethylation.

### Permethylation and MADLI-MS and MS/MS

Permethylation of glycans was carried out as described previously (45). Briefly, *N*-glycans were permethylated by treatment with 0.2 ml of methyl iodide (Merck) in NaOH-DMSO (3 g of NaOH in 2 ml of DMSO) slurry for 15 min at 37 °C with intermittent mixing. Permethylated *N*-glycans were extracted in chloroform, dried under nitrogen, and purified on a Sep-Pak® Classic C_18_ column (Waters) using 3 ml each of 10, 50, and 75% acetonitrile in water. Eluted fractions were lyophilized and redissolved in 20 μl of methanol, mixed with equal volumes of Super-DHB (2,5-dihydroxybenzoic acid; 20 mg/ml in 70% methanol), and spotted on a MALDI plate. MS and MS/MS data were acquired in positive ion mode using an AB SCIEX TOF/TOF 5800 system. Calmix (Applied Biosystems) was used as an internal standard for calibration in both modes. MS/MS collision-induced dissociation was carried out with argon gas at a voltage of 1 kV. Data were acquired using a TOF/TOF Series Explorer (AB SCIEX). Data from 10,000 shots, collected from different areas of the spot (laser intensity, 4500 for MS and 5000–6000 for MS/MS), were summed up and analyzed using Data Explorer software (AB SCIEX). The observed peaks were annotated using GlycoWorkbench software.

### 2-Aminobenzamide labeling and MALDI

*N*-Glycans released from whole-planarian (100 mg wet weight) tissues were labeled with 2-AB (46). Briefly, released glycans were incubated with 10 μl of 2-AB labeling mixture (2.5 mg 2-AB in 50 μl of acetic acid:DMSO (3:7, v/v), 3 mg of sodium cyanoborohydride, and 5 μl of ultrapure water) for 3 h at 65 °C. The reaction was stopped by adding a drop of ammonia; excess borohydride was removed by repeated washing with 200 μl of ethyl acetate. Labeled glycans were recovered in the water phase and dried under vacuum after which they were dissolved in 200 μl of 5% acetonitrile in water and purified using Hypercarb^TM^ solid-phase extraction cartridges (Thermo Scientific). The cartridge was washed successively with 15 ml of 1 m NaOH, ultrapure water, 30% acetic acid, and ultrapure water, respectively. The cartridge was preconditioned with 15 ml of 50% acetonitrile and 0.1% formic acid solution followed by 15 ml of 5% acetonitrile and 0.1% formic acid solution. The 2-AB–labeled sample was passed through the cartridge and washed with 15 ml of 5% acetonitrile solution followed by 15 ml of 15% acetonitrile solution. The bound glycans were eluted in 10 ml of 50% acetonitrile fraction and concentrated. The purified sample was redissolved in 20 μl of 50% methanol, mixed with equal volumes of Super-DHB, and spotted on a MALDI plate. MS and MS/MS data were acquired and analyzed as described earlier under “Permethylation and MADLI-MS and MS/MS.”

### Chemical and enzymatic treatment of N-glycans

#### 

##### Hydrofluoric Acid Treatment

*N*-Glycans were incubated with 50 μl of hydrofluoric acid (48%, v/v) in low-binding microcentrifuge tubes for 24 h at 4 °C and dried under a stream of nitrogen.

##### Enzyme Treatment

β(1–4)-Galactosidase from *A. oryzae* was purified using an SP Sepharose Fast Flow column (GE Healthcare) as described previously (47). *N*-Glycans were incubated with 25 milliunits of enzyme in 10 mm sodium acetate buffer, pH 4.6, for 24 h at 37 °C. Coffee bean α-galactosidase (50 milliunits) and jack bean α-mannosidase (15 milliunits) treatments were carried out in 50 mm ammonium acetate pH 5.0 buffer for 24 h at 37 °C. The β(1–3/6)-galactosidase (P0726S, New England Biolabs) and α-fucosidase (P0748S, New England Biolabs) treatments were carried out for 24 h at 37 °C according to the manufacturer's instructions. Following enzymatic digestion, samples were subjected to Sep-Pak Classic C_18_ cleanup. The purified glycans were recovered in the 5% acetic acid fraction, lyophilized, permethylated (except those treated with α-mannosidase), and subjected to MALDI-TOF and TOF/TOF MS analysis. The α-mannosidase–treated sample was subjected to 2-AB labeling before MALDI-TOF and TOF/TOF MS analysis.

### Determination of monosaccharide composition and linkage of N-glycans using GC-MS

Monosaccharide composition and linkage of glycans were determined using GC-MS of partially methylated alditol acetates (PMAAs) (48). *N*-Glycan release and permethylation were carried out as described earlier under “Permethylation and MADLI-MS and MS/MS.” Permethylated glycans were hydrolyzed (2.5 m trifluoroacetic acid for 4 h at 100 °C), reduced (10 mg of sodium borodeuteride in 2 m ammonium hydroxide for 2 h at room temperature), and acetylated in 400 μl of acetic anhydride:pyridine (3:1, v/v) for 1 h at 100 °C. The PMAAs formed were dissolved in 50 μl of chloroform of which 2 μl were used for analysis. GC-MS of PMAAs was carried out in a Clarus SQ 8C GC/MS (PerkinElmer Life Sciences) using an RTX-5 fused silica column of length 30 m and internal diameter 0.32 mm (Restek, Corp.) with helium as the carrier gas. The temperature of the oven was maintained at 40 °C for 1.5 min, ramped up to 130 °C at 40 °C/min and then to 290 °C at 8 °C/min, and maintained at 290 °C for 5 min (49).

### Sequence analysis

To identify putative GALT-1 sequences in *S. mediterranea*, amino acid sequences of GALT-1 from *C. elegans* (accession number NP_504545.2) and its avian homolog from *C. livia* (accession number FJ971845.1/ADC84389.1) were used as query sequences for a sequence similarity search using BLASTp against the PlanMine database (23). All entries in the GT-92 glycosyltransferase family listed in the CAZy database (50) and the two putative GALT-1 sequences from *S. mediterranea* obtained from the sequence search were aligned using MUSCLE (51). Phylogenetic analysis was performed using the maximum likelihood method based on the JTT matrix-based model (52) for 1000 bootstraps using MEGA7 (53). Domain organization was ascertained using SMART (54). The distribution of *GALT-1* in different cell types was ascertained using the SCS Whitehead database (24).

### RNA isolation, cDNA synthesis, and PCRs

Total RNA was isolated from planarians using TRIzol reagent (Invitrogen) according to the manufacturer's instructions. First-strand cDNA synthesis was carried out with 5 μg of total RNA and 1 μg of oligo(dT) (Invitrogen) using the Superscript III first-strand synthesis kit (Invitrogen). PCR amplification was performed using TaKaRa LA *Taq* (R002M) at an annealing temperature of 52 °C. PCR products were gel-extracted using the Wizard® SV Gel and PCR Clean-Up System (Promega) and cloned into the pCR^TM^II-TOPO® vector using the TOPO TA cloning kit (Invitrogen) according to the manufacturer's instructions. The plasmid was isolated from transformed clones using the QIAprep® Miniprep kit (Qiagen) and verified for inserts using in-house Sanger sequencing. The primer sequences used were: *Smed-galt-1-1*: forward, CGTCTGAAACTCTCCAATGGAC; reverse, TGAAACCAAACATTCCATCGCA; *Smed-galt-1-2*: forward, TCGGACAAGAAACAGTTACAGA; reverse, TTTCCCCTAAGCCATCCCAG.

### Whole-planarian in situ hybridization

A digoxigenin (DIG)-labeled antisense riboprobe was synthesized from the linearized plasmid using Sp6 polymerase (Roche Applied Science) and a DIG RNA labeling kit (Roche Applied Science) according to the manufacturer's instructions. Formaldehyde-based whole-planarian *in situ* hybridization was carried out as described previously (17). Briefly, planarians were killed by treatment with 5% *N*-acetylcysteine, fixed in 4% formaldehyde for 40 min, dehydrated in 100% methanol, and bleached with 6% H_2_O_2_ in methanol under white light. Hybridization was carried out at 55 °C for 16 h. Following hybridization, planarians were washed thrice with 2× SSC buffer (saline-sodium citrate buffer, pH 7.0, containing 0.1% Triton X-100) followed by three washes with 0.2× SSC at 55 °C for 20 min each. Samples were blocked in MABT buffer (100 mm maleic acid, 150 mm NaCl, and 0.1% Tween 20, pH 7.5) containing 5% horse serum and Western blocking reagent (Roche Applied Science) for 2 h at room temperature followed by incubation with alkaline phosphatase–conjugated anti-DIG Fab (1:2000; Roche Applied Science) in blocking buffer for 12 h at 4 °C. Color was developed using nitroblue tetrazolium/5-bromo-4-chloro-3-indolylphosphate as the chromogen (1:30; Roche Applied Science) in AP buffer (100 mm Tris-HCl, pH 9.5, 100 mm NaCl, 50 mm MgCl_2_, 0.1% Tween 20, and 2% polyvinyl alcohol) by incubating in the dark for 2 h at room temperature.

### RNA interference

Knockdown of target genes was performed using double-stranded RNA–meditated interference (55). dsRNA was prepared using T7 RNA polymerase (Roche Applied Science). Each 25-μl reaction contained 2.5 μg of template, 8 mm rNTPs (New England Biolabs), 2 μl of T7 polymerase, 1 μl of RNaseOUT (Invitrogen), and nuclease-free water. The contents were incubated at 37 °C for 16 h and treated with DNase I for 1 h at 37 °C. The dsRNAs produced from four such reactions were pooled and recovered by precipitation with 0.6 m lithium chloride and 75% ethanol. RNAi was performed by injecting dsRNA for 5 consecutive days (280 nl each day) using a Nanoject II injector (Drummond Scientific Co.). The dsRNA prepared from a GFP plasmid was used as a negative control. Phenotypes were scored on the 6th day. Knockdown efficiency was verified using real-time quantitative PCR. Real-time quantitative PCR was carried out in a 384-well plate using the Maxima SYBR Green/ROX Master Mix (Thermo Scientific) on a CFX384 Touch^TM^ real-time PCR detection system (Bio-Rad). A technical triplicate of three biological trials was performed, and the mean threshold value of each gene was normalized with *S. mediterranea* actin as described previously (56).

### Imaging

Whole-mount *in situ* hybridization and knockdown phenotypes were imaged on an Olympus SZX 16 stereomicroscope using cellSens Dimension software. Acquired images were analyzed using ImageJ software.

## Author contributions

S. S. P., D. P., and R. S. conceptualization; S. S. P. and P. B. data curation; S. S. P., P. B., and D. P. investigation; S. S. P., P. B., and D. P. methodology; S. S. P. and D. P. writing-original draft; S. S. P., P. B., D. P., and R. S. writing-review and editing; P. B. and R. S. formal analysis; P. B. validation; R. S. funding acquisition.

## Supplementary Material

Supporting Information

## Footnotes

3 The abbreviations used are:

PNGase A: peptide:*N*-glycosidase A
F_1_H*_n_*N_2_: Fuc_1_Hex_2–9_HexNAc_2_
Man-2: -3, -4, -5, -6, and -7, Man_2_GlcNAc_2_, Man_3_GlcNAc_2_, Man_4_GlcNAc_2_, Man_5_GlcNAc_2_, Man_6_GlcNAc_2_, and Man_7_GlcNAc_2_
CCM: core chitobiose modification
PMAA: partially methylated alditol acetate
GalFuc: Galβ1–4Fuc motif
2-AB: 2-aminobenzamide
SCS: single cell sequencing
dsRNA: double-stranded RNA
DHB: 2,5-dihydroxybenzoic acid
DIG: digoxigenin
SMED/*Smed*: *S. mediterranea*
Man: mannose
Gal: galactose
Fuc: fucose
HexNAc: *N*-acetylhexosamine.
