# Supplementary material for: Identification of multiple isomeric core chitobiose–modified high-mannose and paucimannose N-glycans in the planarian Schmidtea mediterranea
Source: J Biol Chem. 2018 Feb 23;293(18):6707–20. doi: 10.1074/jbc.RA117.000782 (PMC5936828; doi:10.1074/jbc.RA117.000782)
Supplement: Supporting Information [file supp_293_18_6707__index.html]

Identification of multiple isomeric core chitobiose-modified high mannose and paucimannose N-glycans in the planarian Schmidtea mediterranea — Unusual core-chitibiose modified N-glycans — Identification of multiple isomeric core chitobiose–modified high-mannose and paucimannose N-glycans in the planarian Schmidtea mediterranea — Unusual core chitobiose–modified N-glycans — Supporting Information 

# Identification of multiple isomeric core chitobiose–modified high-mannose and paucimannose *N*-glycans in the planarian *Schmidtea mediterranea*

## Supporting Information

- Supplementary data - Supplementary Figures and tables
