# Supplementary material for: Identification of multiple isomeric core chitobiose–modified high-mannose and paucimannose N-glycans in the planarian Schmidtea mediterranea
Source: J Biol Chem. 2018 Feb 23;293(18):6707–20. doi: 10.1074/jbc.RA117.000782 (PMC5936828; doi:10.1074/jbc.RA117.000782)
Supplement: Supporting Information [file supp_RA117.000782_133663_3_supp_79161_p4gbgg.docx]

**Supporting Information**

Identification of multiple isomeric core chitobiose-modified high mannose and paucimannose N-glycans in the planarian *Schmidtea mediterranea*

**Sabarinath Peruvemba Subramanian^1^, Ponnusamy Babu^2$^, Dasaradhi Palakodeti^1^, and Ramaswamy Subramanian^1#^**

From the ^1^Institute for Stem Cell Biology and Regenerative Medicine (inStem), GKVK Post Office, Bellary Road, Bangalore -560065, Karnataka, India; ^2^Glycomics and Glycoproteomics Facility, Centre for Cellular and Molecular Platforms (C-CAMP), GKVK Post Office, Bellary Road, Bangalore -560065, Karnataka, India.

**Running title:** Unusual core chitobiose-modified N-glycans

^#^**To whom correspondence should be addressed:** Ramaswamy Subramanian: ^1^Institute for Stem Cell Biology and Regenerative Medicine (inStem), GKVK Post Office, Bellary Road, Bangalore -560065

Email: [*ramas@instem.res.in*](mailto:ramas@instem.res.in)*.* Phone: Office +91 8067176820, Lab +91 08067176808.

^$^**Co-corresponding author:** Ponnusamy Babu**:** Email: [*ponbabu@gmail.com*](mailto:ponbabu@gmail.com)*.* Phone: +91 9739012156

**List of supplementary materials**

Figure S1: MALDI-TOF/TOF MS spectra of complex and hybrid structures

Figure S2: MALDI-TOF MS spectra of chemical and enzymatic digestion

Figure S3: GC-MS monosaccharide and linkage analysis of partially methylated alditol acetates (PMAAs) of N-glycans in *S. mediterranea*

Figure S4: MALDI-TOF/TOF MS spectra of singly charged monosodiated [M+Na]^+^ 2-AB labeled N-glycan of m/z 1595

Figure S5: MALDI-TOF MS spectra of α-mannosidase treated N-glycans of *S. mediterranea*

Figure S6: Phylogenetic tree of GT-92 family of enzymes

Figure S7: Knockdown of *Smed-galt-1* affects tissue homeostasis

Figure S8: MALDI-TOF MS spectra of N-glycans from *Dugesia species* (Indian strain IN 06)

**Figure S1**

**
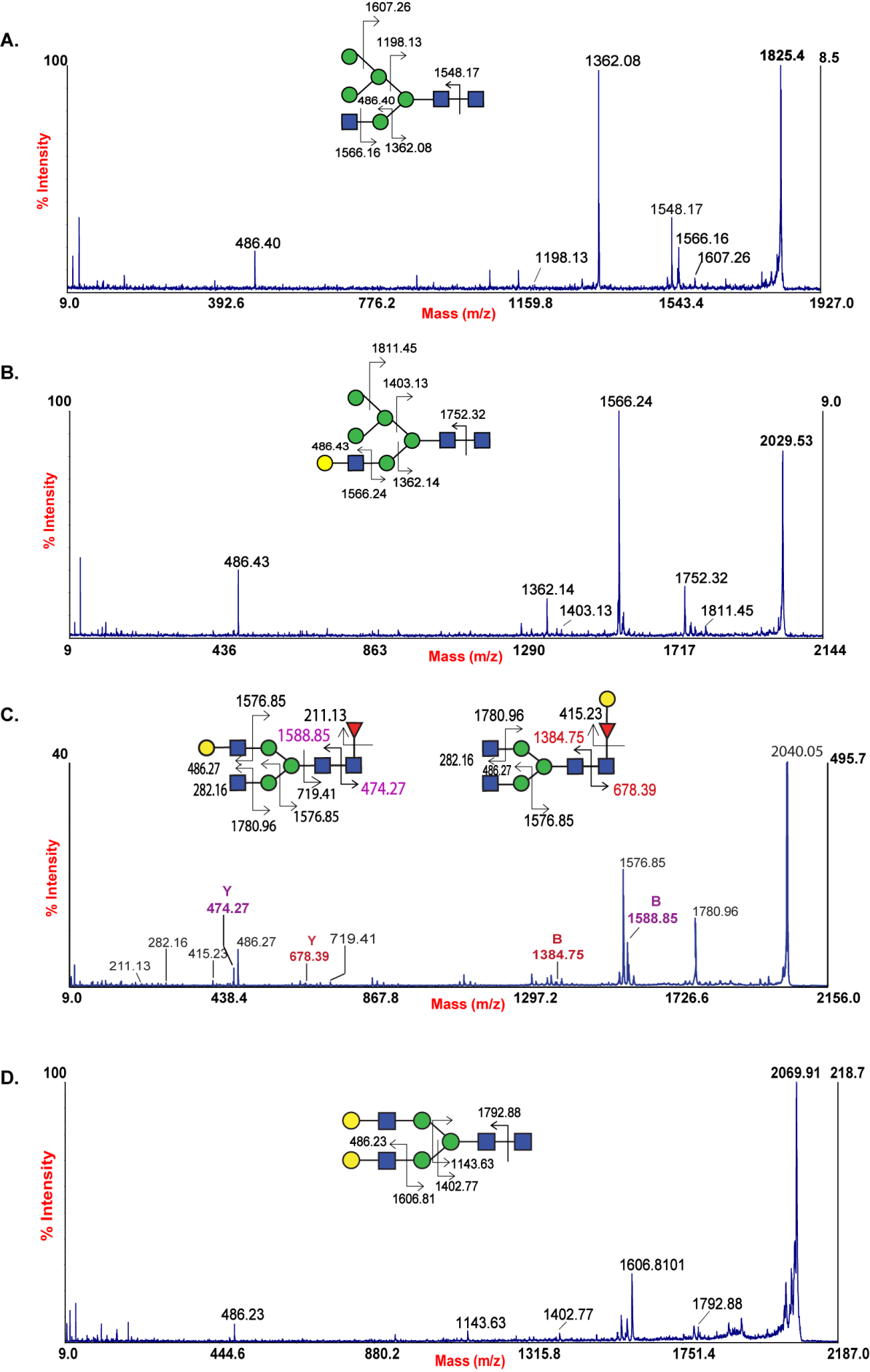
**

**
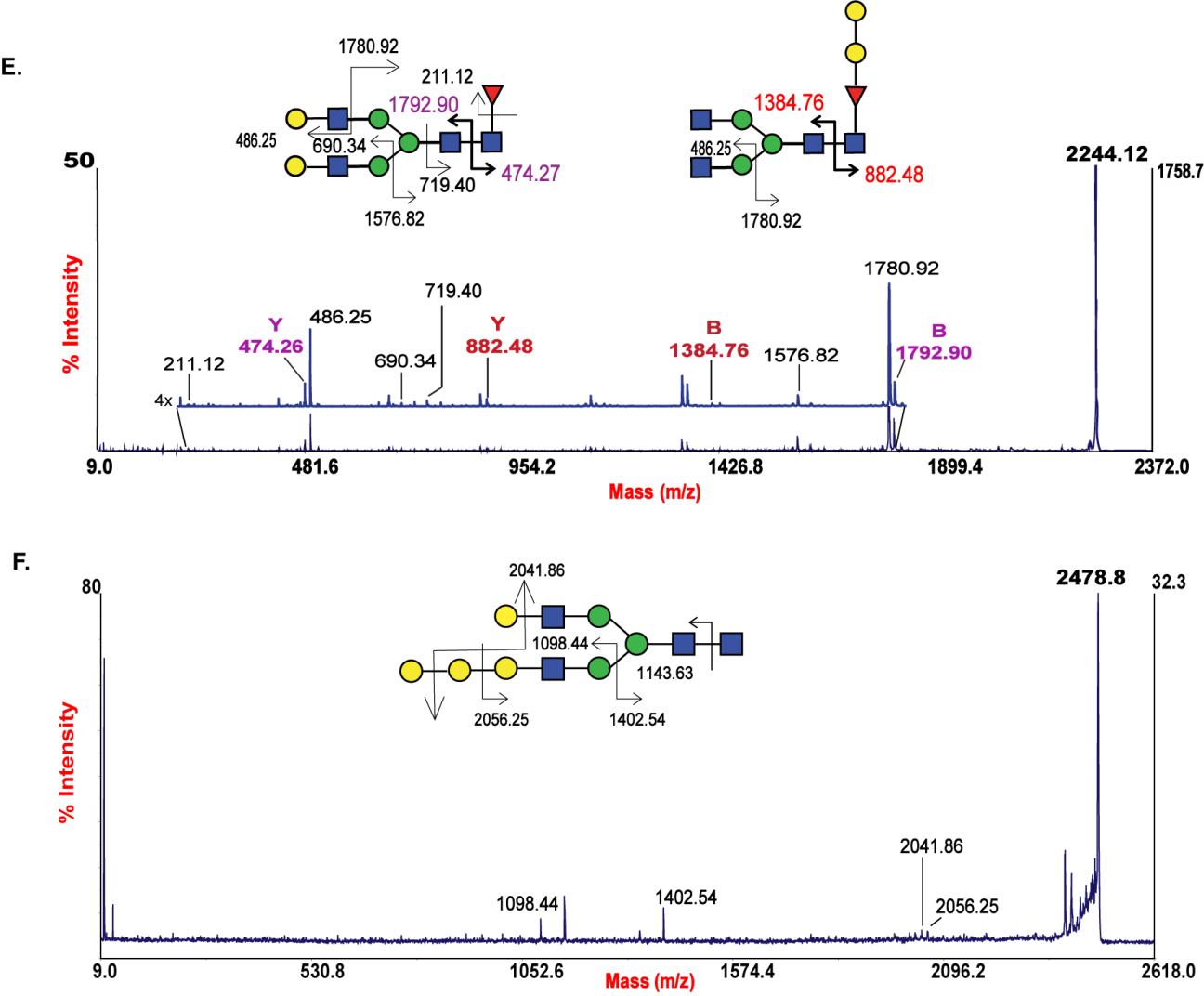
**

**Fig. S1: MALDI-TOF/TOF MS spectra of complex and hybrid structures**

PNGase A released glycans were permethylated and subjected to MALDI-TOF/TOF MS analysis. Data represents MS/MS spectra of singly charged monosodiated [M+Na]^+^ permethylated N-glycans of *m/z* A) 1825, B) 2029, C) 2040, D) 2070, E) 2240, and F) 2478 obtained in 50% acetonitrile fraction. B- and Y- fragment ions obtained are represented. (A-B) Fragment ions from *m/z* 1824 and 2029 indicate presence of hybrid structure. (C-F) The characteristic fragment ions obtained show presence of complex-type structures with and without core-fucose. For m/z 2040 and 2244, major fragment ions (B-ion 1588 and Y-ion 474 in 2040; B-ion 1792 and Y-ion 474 in 2244) obtained in MS/MS spectra reveal that complex bi-antennary structure with core-fucose is the most predominant glycoform. In addition to complex type glycans, the presence of B-ion 1384 and Y-ion 678 at *m/z* 2040 and B-ion 1384 and Y-ion 678 at *m/z* 2244 indicate presence of mono and di-galactosylated core-fucose, as minor constituents. Extensions in complex biantennary glycans were seen at m/z 2478. Additional isomers with fragment ion m/z 1157 Hex4HexNAc2 and m/z 1343 Hex3HexNAc2 were also seen at m/z 2478.

**Figure S2**

**
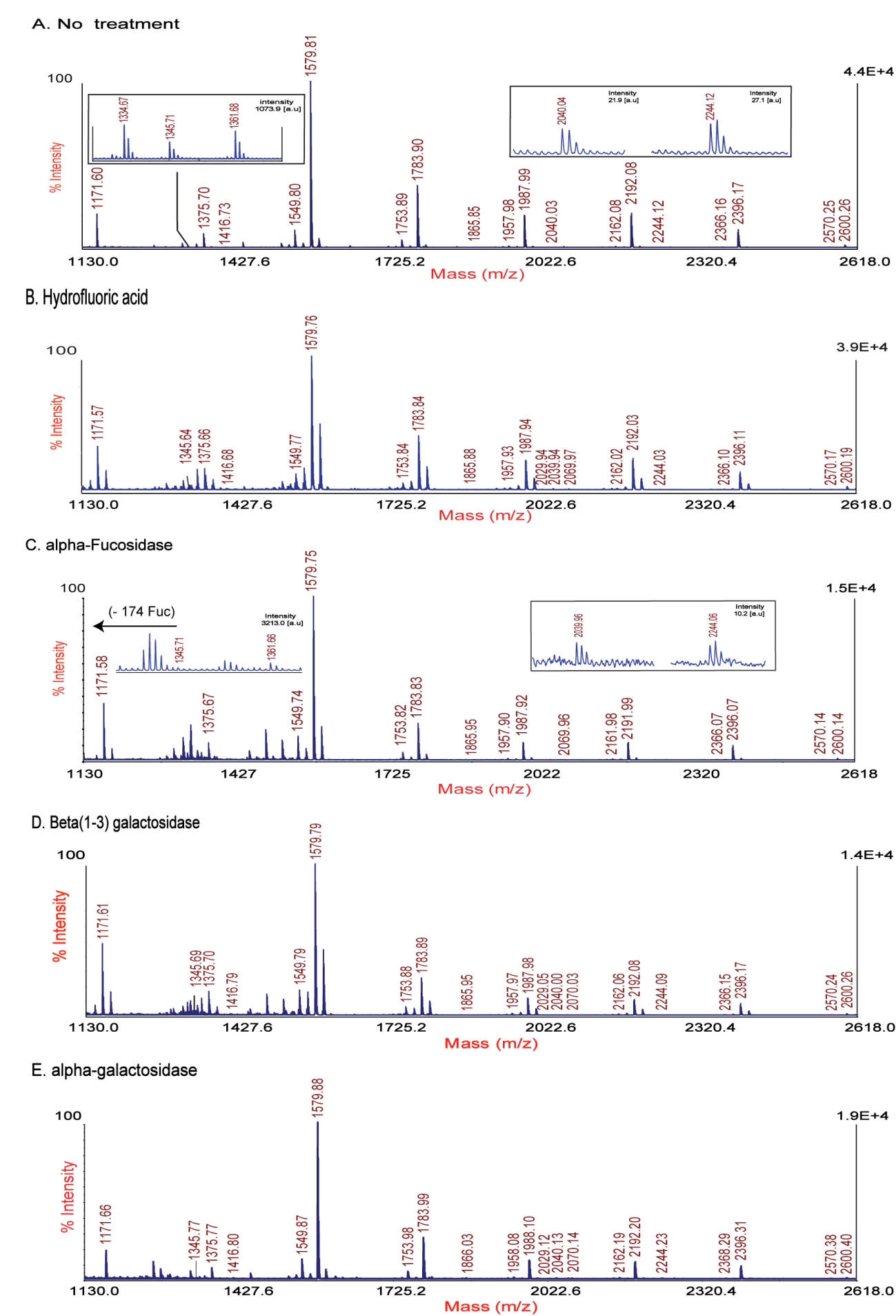
**

**Fig. S2: MALDI-TOF MS spectra of chemical and enzymatic digestion**

N-glycans released by PNGase A treatment were subjected to chemical and enzymatic digestion. The hydrolyzed products thus obtained were permethylated and analyzed using MALDI-TOF MS. Data represents MS spectra of singly charged monosodiated [M+Na]^+^ permethylated N-glycans obtained in 50% acetonitrile fraction. (Fig.S2 A) MS spectra of untreated N-glycans (control). (Fig.S2 B and C) MS spectra of hydrofluoric acid and α-fucosidase treated samples. MS spectra reveal resistance to both hydrofluoric acid and α-fucosidase digestion. These results suggest absence of α1-2 and α1-3 linked fucose and indicate that fucose is 1-6 linked core-GlcNAc moieties. Resistance to α-fucosidase indicates capping of core-fucose. m/z 1345 was susceptible to fucosidase digestion. (Fig.S2 D and E) MS spectra of β1-3/6 galactosidase and α-galactosidase treated samples. Treatment with both α-and β1-3/6 galactosidase was inefficient in hydrolyzing the core-modified structures.

**Figure S3**

**
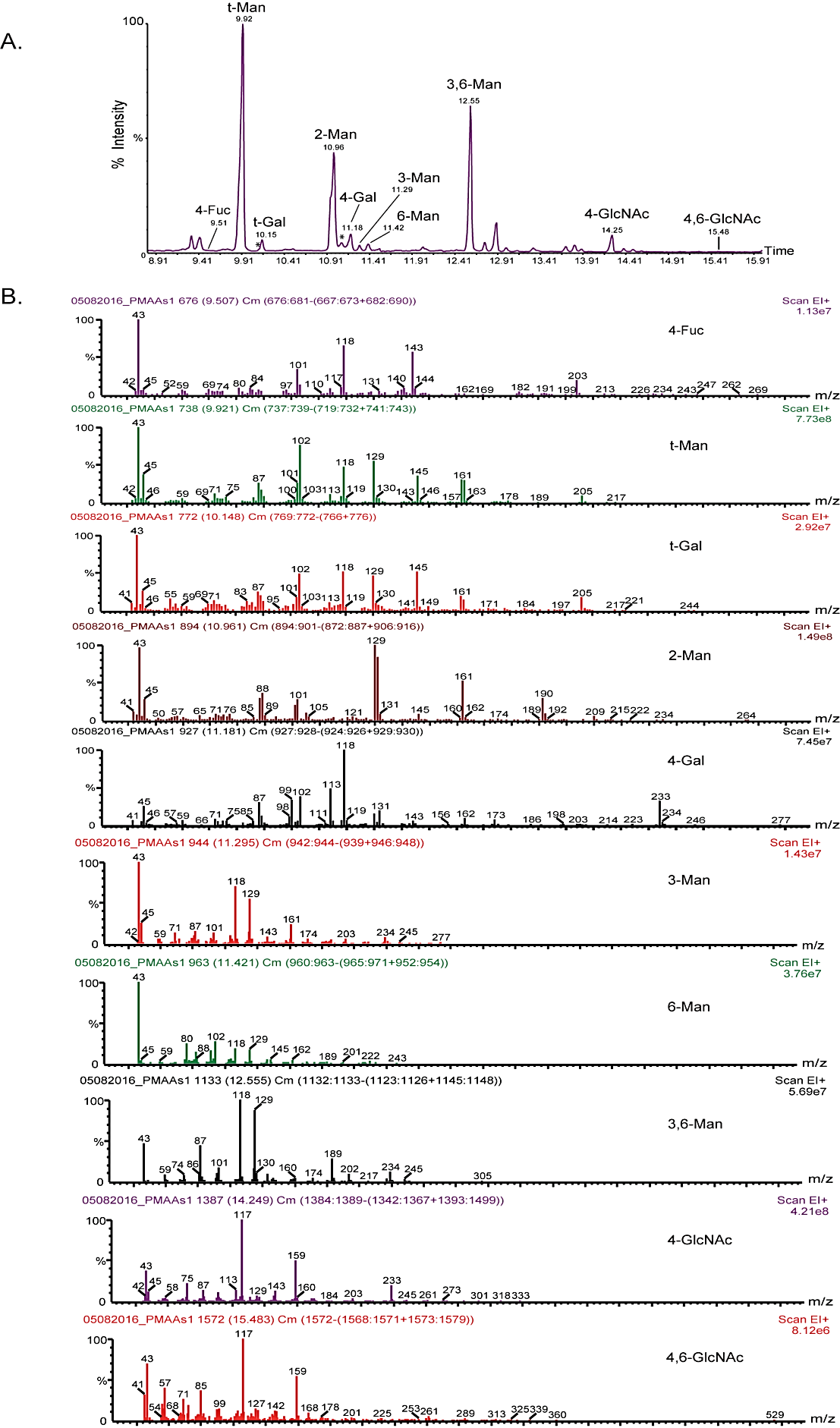
**

**Fig. S3:** **GC-MS monosaccharide and linkage analysis of partially methylated alditol acetates (PMAAs) of N-glycans in *S. mediterranea***

N-glycans released with PNGase A treatment were permethylated, hydrolyzed, reduced, and acetylated as described in methods. PMAAs thus formed were subjected to GC-MS analysis.

(A) GC chromatogram of PMAAs. Monosaccharide composition and their linkage were determined based on the retention times and characteristic electron impact spectra. Monosaccharide derivatives obtained are indicated in the chromatogram. Terminal fucose was undetected. (*) Glucose contaminant.

(B) Electron impact spectra of PMAAs. Structural assignment of monosaccharide and its linkage was made based on the characteristic diagnostic ions.

**Figure S4**

**
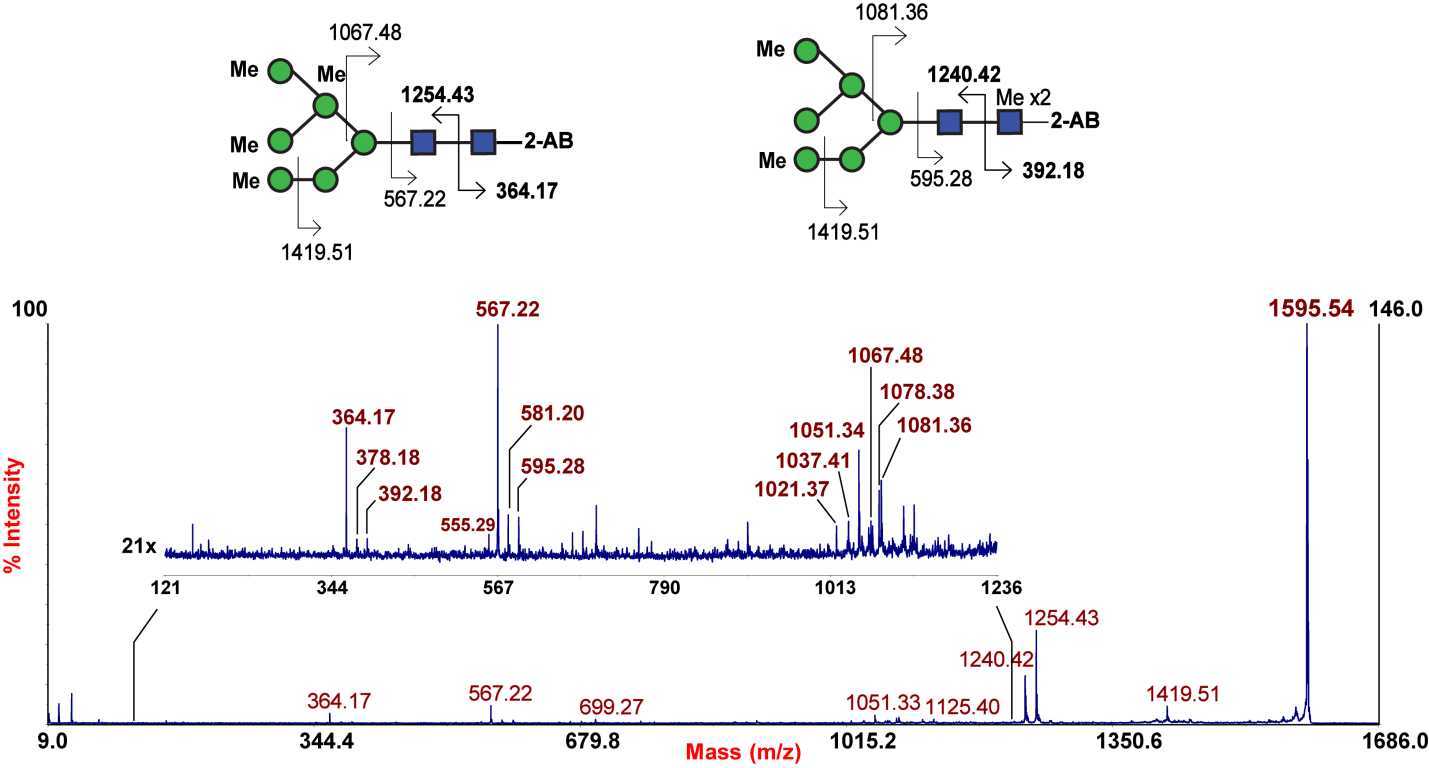
**

**Fig. S4: MALDI-TOF/TOF MS spectra of singly charged monosodiated [M+Na]^+^** **2-AB labeled N-glycan of m/z 1595**

PNGase A released glycans were subjected to 2-AB labeling and MS analysis. Spectra represent two other glycomers (apart from the ones shown in Fig.5A) with different methylation profile. A glycoform with methylation at the α1-6 mannose arm in mannose termini and another with dimethylated core-GlcNAc are presented.

**Figure S5**


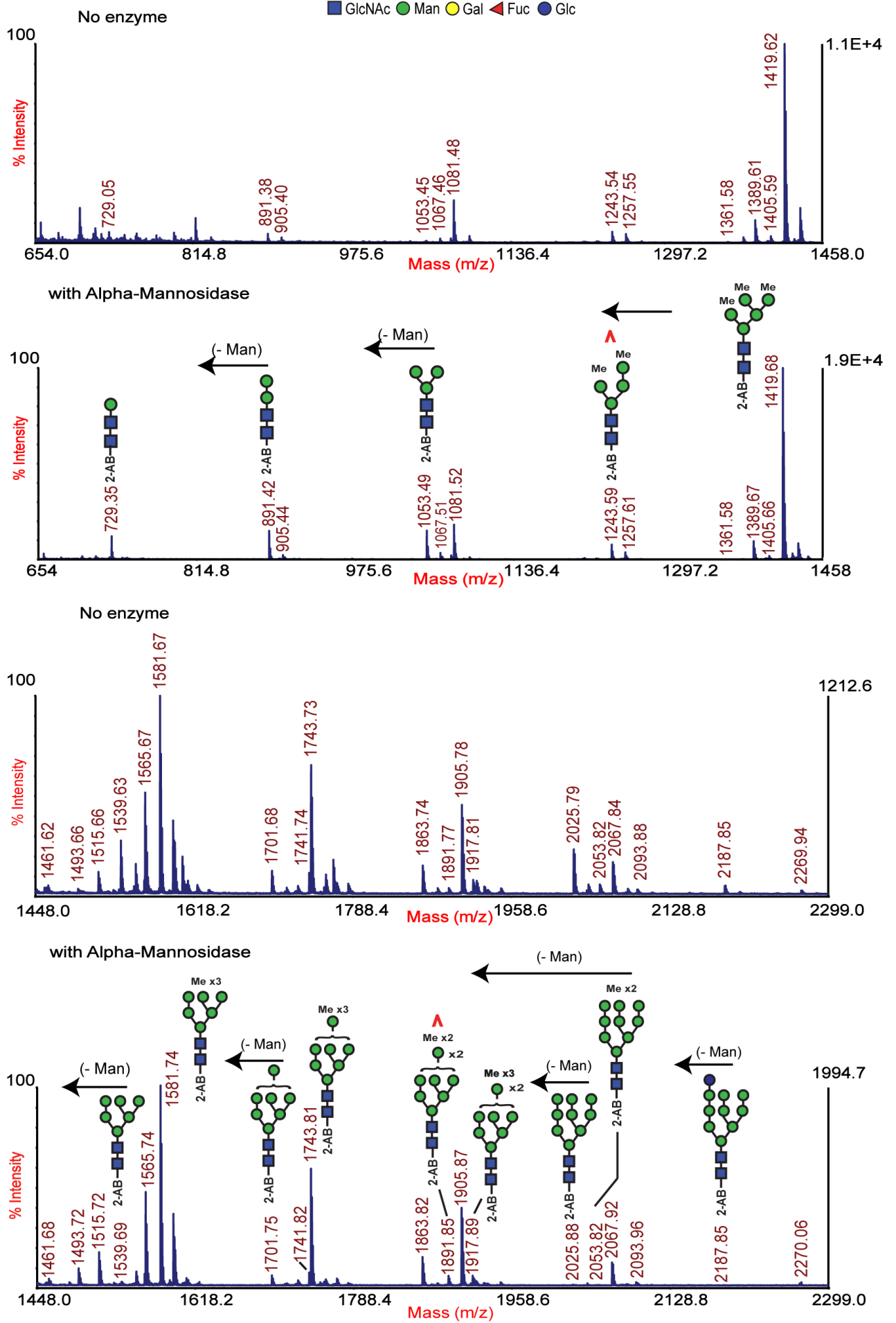


**Fig. S5: MALDI-TOF MS spectra of α-mannosidase digested N-glycans of *S. mediterranea***

PNGase A released glycans treated with or without jack bean α-mannosidase were labeled with 2-AB and analyzed using MALDI-TOF MS. Data represents MS spectra from the 50% acetonitrile fraction of 2-AB labeled glycans obtained from Hypercarb SPE cartridge. Molecular ions indicated are C^12^ mono-isotopic peaks of singly charged monosodiated [M+Na]^+^ structures. Structures were assigned based on putative composition, MS/MS, and biosynthetic knowledge. Monosaccharide moieties shown outside brackets are not unequivocally defined. Methylated glycans were resistant to mannosidases and showed no change in the intensity of peaks when compared to controls. Non-methylated and partially methylated N-glycans showed considerable reduction in the intensity of peaks (← indicates loss of mannose between original glycan and its product). The reduction in peaks was followed by a concomitant increase in the preceding mass (**^**). Core chitobiose-modified glycans at *m/z* 1389, 1565, 1741, 1917, 2093, and 2270 were resistant to mannosidase digestion.

**Figure S6**

**
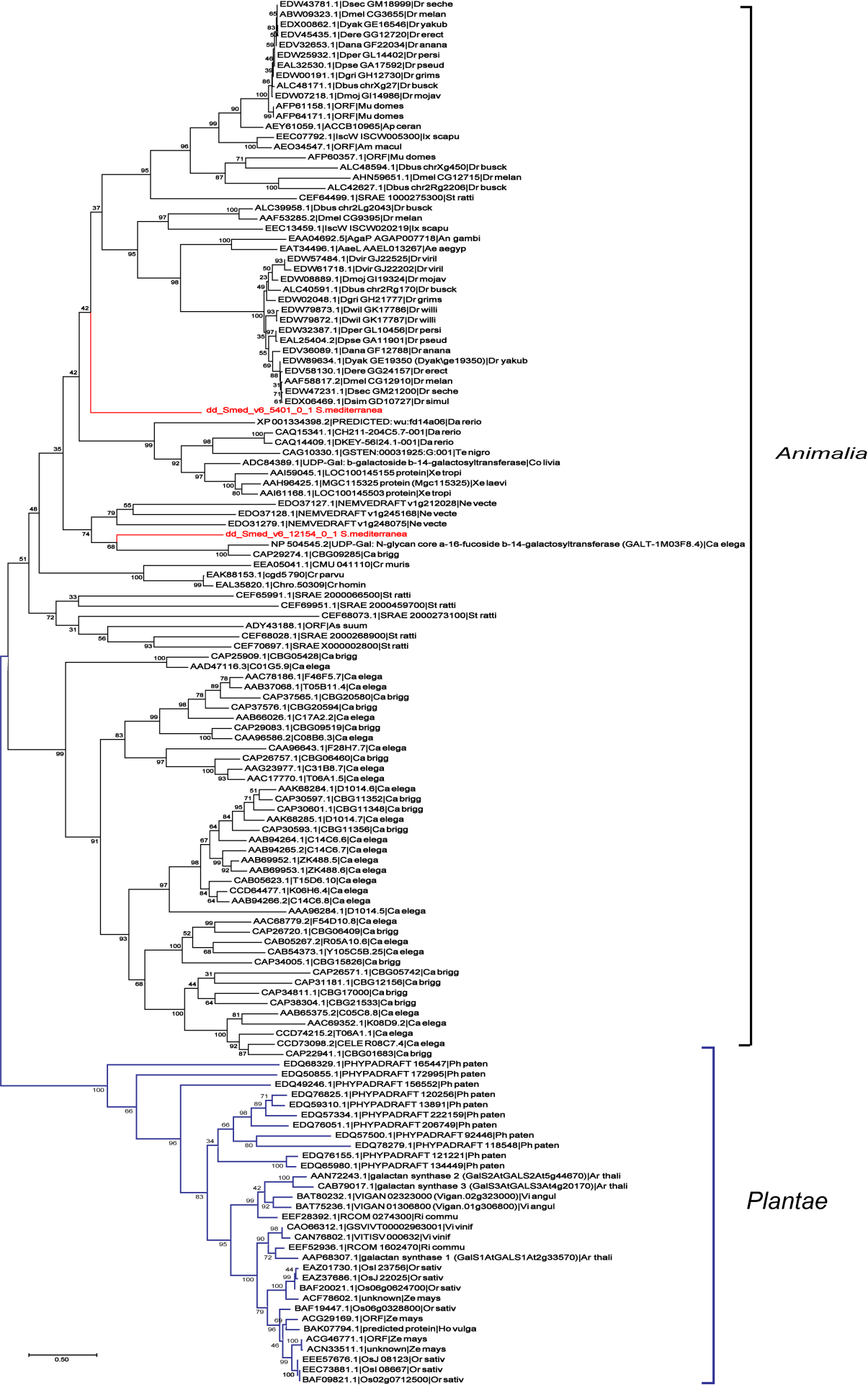
**

**Fig. S6: Phylogenetic tree of GT-92 family of enzymes**

The evolutionary history was inferred using the Maximum Likelihood method based on the JTT matrix-based model. The tree with the highest log likelihood (-56579.88), the Gene bank accession number, protein name in CAZy database, and the name of the organism are shown. The percentage of trees in which the associated taxa clustered together is shown next to the branches. Initial tree(s) for the heuristic search were obtained automatically by applying Neighbor-Join and BioNJ algorithms to a matrix of pairwise distances estimated using a JTT model and then selecting the topology with superior log likelihood value. The analysis involved 136 amino acid sequences. All positions with less than 90% site coverage were eliminated. That is, fewer than 10% alignment gaps, missing data, and ambiguous bases were allowed at any position. There were a total of 322 positions in the final dataset.

**Figure S7**

**
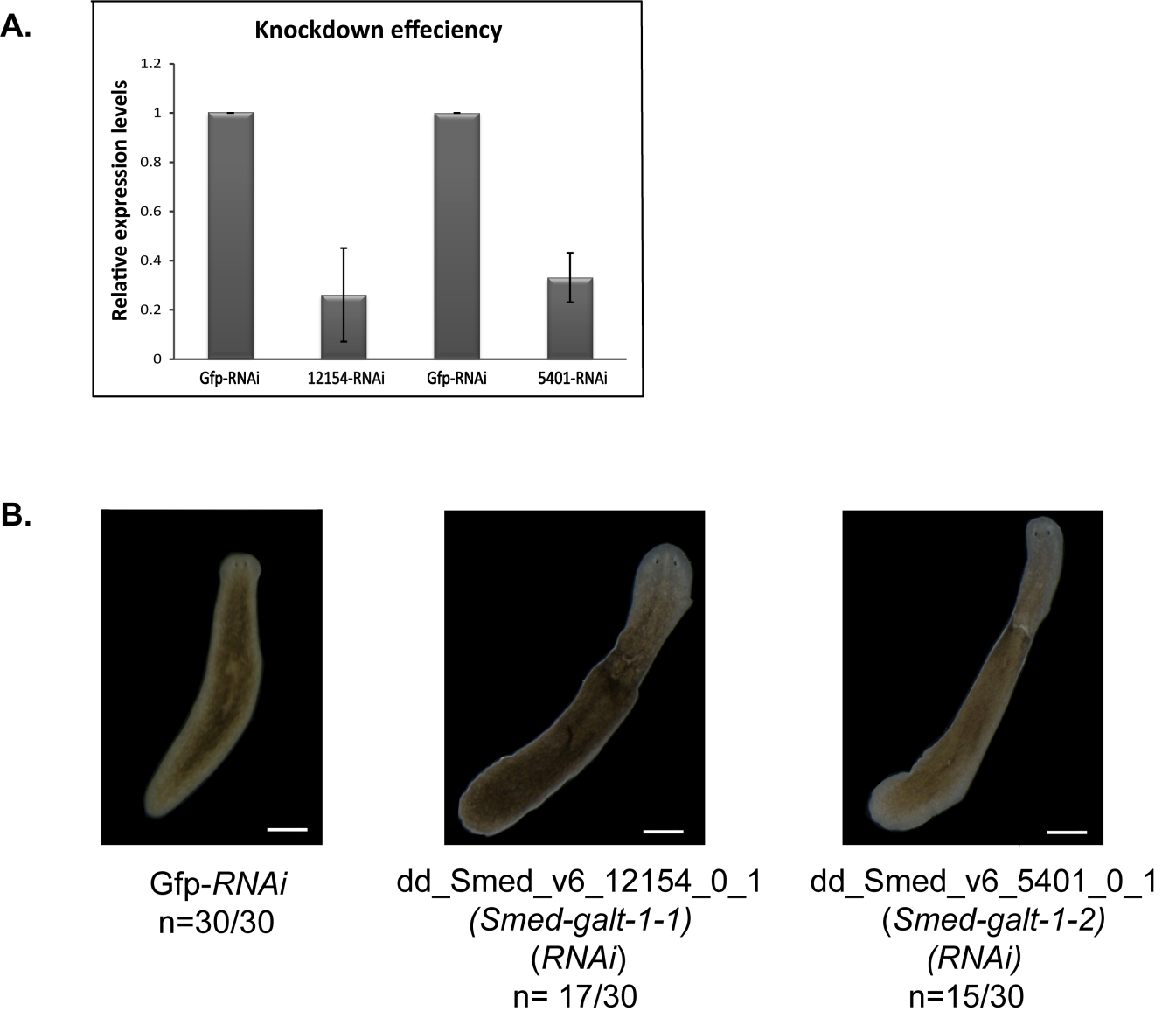
**

**Fig. S7: Knockdown of *Smed-galt-1* affects tissue homeostasis**

(A) Real-time qPCR analysis of *Smed-galt-1*. qPCR was performed on day 6 after 5 consecutive days of dsRNA injection *(Gfp-control, 12154, and 5401)*. RNAi phenotype worms showed reduction in the expression (60-70%) of dd_Smed_v6_12154 and dd_Smed_v6_5401, respectively, compared to Gfp-RNAi controls. Data represent mean ± standard deviation of three biological trials carried out in triplicates. Mean threshold values were normalized to actin and relative expression levels of candidate genes are represented in relative units. (B) Representative images of RNAi phenotypes observed in intact worms. Gfp-RNA*i* – control showed no morphological change, dd_Smed_v6_12154 and dd_Smed_v6_5401 RNA*i* presented *stick and stretch* phenotype. Scale bar 200 µm.

**Figure S8**

**
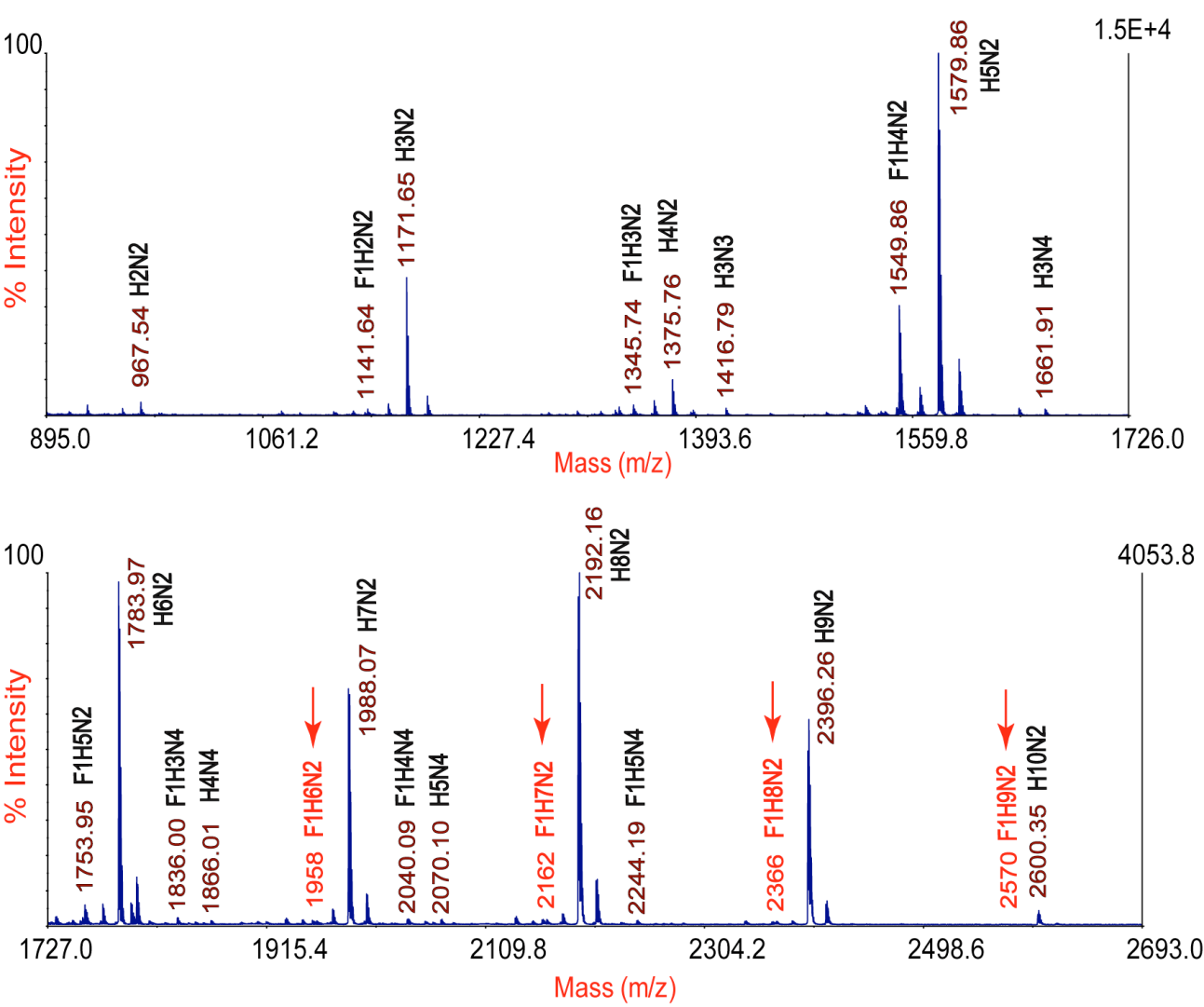
**

**Fig. S8: MALDI-TOF MS spectra of N-glycans from *Dugesia species* (Indian strain IN 06)**

N-glycans from the Indian strain of *Dugesia* (IN 06) isolated with PNGase A treatment were permethylated and analyzed using MALDI-TOF MS. Data represent MS spectra of the 50% acetonitrile fraction of permethylated N-glycans obtained from C18 cartridge clean up. Molecular ions indicated are C^12^ mono-isotopic peaks of singly charged monosodiated [M+Na]^+^ structures. Putative compositions of each molecular ion are indicated (F-fucose, H-hexose and N-hexosamine). Molecular ions highlighted in red represent absence of *m/z* 1958, 2162, 2366, and 2570 in the Indian strain.
